# The alpha tubulin acetyltransferase *atat-2* genetically interacts with *klp-4* in *C. elegans*

**DOI:** 10.17912/micropub.biology.001536

**Published:** 2025-04-11

**Authors:** Claire E. Reist, Michael D. Webb, Cortlen M. Mathews, Jay N. Pieczynski

**Affiliations:** 1 Department of Pathology and Laboratory Medicine, University of North Carolina at Chapel Hill; 2 Department of Biology, Rollins College

## Abstract

Microtubules dynamics are in part regulated by post-translational modification, including acetylation. Little is known about the relationship between microtubule acetylation status and how this affects kinesin function, especially *in vivo* . Using a series of aldicarb sensitivity assays in *C. elegans* where we combined pharmacological manipulation of microtubule dynamics with genetic approaches, we demonstrate a specific genetic interaction between the alpha tubulin acetyltransferase *atat-2* and the kinesin motor *klp-4* . Our work highlights interactions between kinesin activity and the tubulin code *in vivo and* lays the foundation of future work on these two parallel, yet related processes in cells.

**Figure 1. klp-4 specifically and genetically interacts with atat-2:**
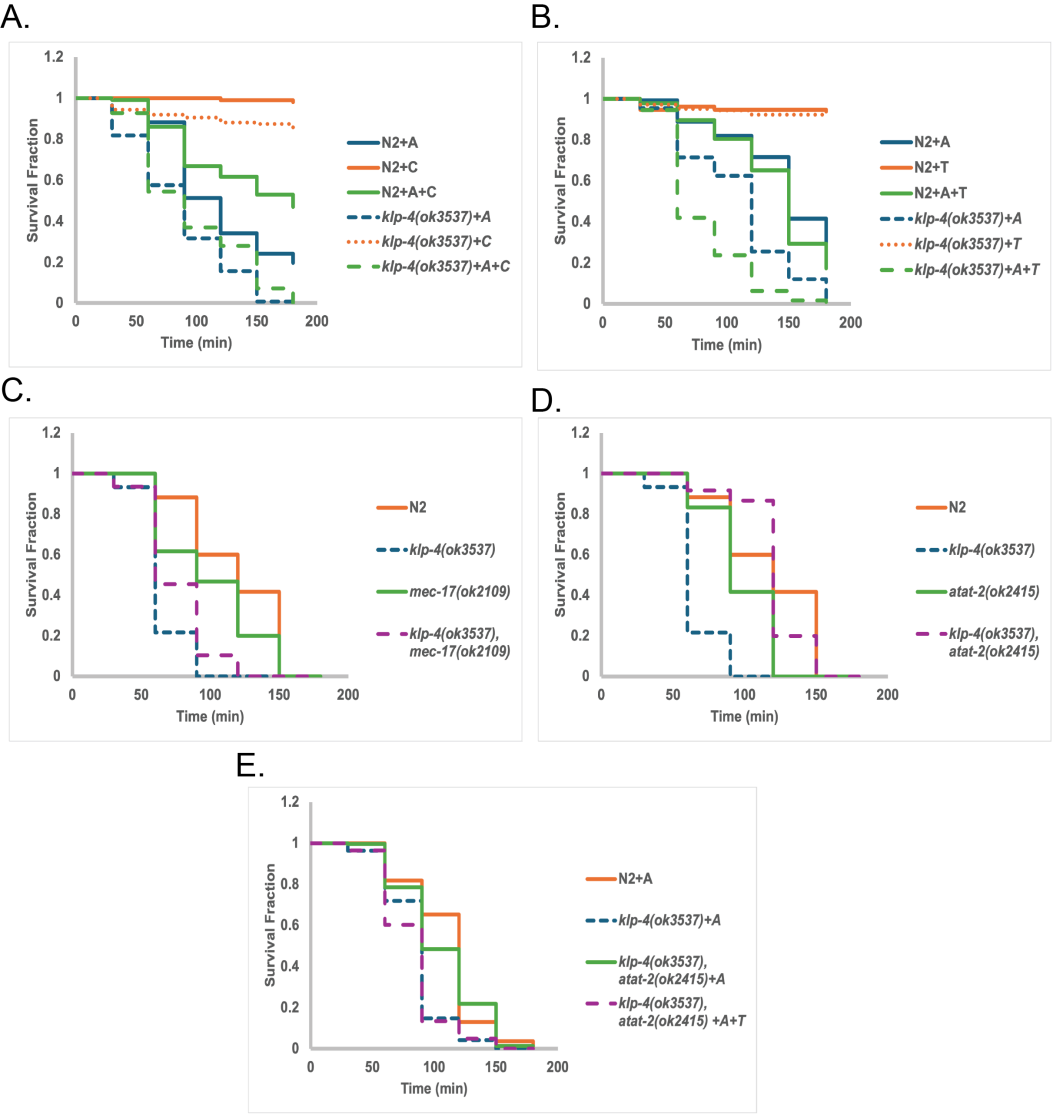
Aldicarb analysis for all genotypes and treatments indicated. All experiments were completed as triplicate triplicates. All assays were analyzed using log-rank statistical tests on aggregate samples to determine significance. Treatments: aldicarb only (+ A), colchicine only (+ C), or aldicarb and colchicine (+A+C), taxol only (+T), aldicarb and taxol (+A +T). A. Aldicarb assays of N2 and *klp-4 ( ok3537 )* with and without colchicine treatment. B. Aldicarb assays of N2 and *klp-4 ( ok3537 )* with and without taxol treatment. C. Aldicarb assays of *klp-4 ( ok3537 ), mec-17 ( ok2109 )* single or double mutants. D. Aldicarb assay of *klp-4 ( ok3537 ), atat-2 ( ok2415 )* single or double mutants. E. Reversion of aldicarb hypersensitivity phenotype by treatment of *klp-4 ( ok3537 ); atat-2 ( ok2415 )* double mutants with taxol.

## Description

Interactions between the microtubules and their associated kinesin motor proteins are essential to proper cell physiology. Two key features of microtubules are their (+/-) polarity and their dynamic instability whereby they grow and shrink dynamically from their (+) ends. Kinesins are processive motor proteins that transport varied cargoes along these microtubules, often in the (+) end direction. When combined, the polarized dynamic instability of microtubules and processive movement of kinesins allow for cells to respond rapidly to constantly changing stimuli. Thus, the functional dynamics of microtubules and kinesins are inherently linked. The dynamic instability of microtubules is primarily controlled by three separate but related factors: tubulin isotype, conformation changes to (+) ends, and interactions with microtubule associated proteins (MAPs). MAPs can function to quickly stabilize/destabilize the microtubule structure either through direct binding or enzymatic post-translational modifications (PTMs, (Janke & Magiera, 2020)). Microtubule stabilization by PTM is especially important in static cellular structures, like cilia or neuronal projections, where stabilized microtubules have essential roles in neuronal outgrowth during development and the establishment and maintenance of sustained signaling during maturity (Eshun-Wilson et al., 2019; Moutin et al., 2020; Nekooki-Machida & Hagiwara, 2020). One of the major stabilizing PTMs is α-tubulin lysine 40 (K40) acetylation (Xu et al., 2017). Some kinesin motor proteins demonstrate preference for acetylation-stabilized microtubules, suggesting cellular crosstalk between the tubulin acetyltransferase/deacetylase MAPs and kinesin function (Andreu-Carbó et al., 2024). To probe any potential interactions between kinesins and microtubule acetylation *in vivo* we utilized the well described *C. elegans* nervous system as model.

To assess the *in vivo* relationship between kinesin motors and microtubule acetylation, we utilized the *C. elegans* strain RB2546 that contains the *klp-4 ( ok3537 )* allele. KLP-4 is member of the highly processive Kinesin-3 superfamily, which is homologous to human KIF13A and KIF13B (Hirokawa et al., 2009). We previously described the *klp-4 ( ok3537 )* variant as having a large, in frame deletion likely resulting in a protein product that is missing a portion of its cargo-binding and/or autoinhibitory domain. The effect of the *klp-4 ( ok3537 )* mutation is predicted to be constitutively active version of the motor (Magaletta et al., 2019; Soppina et al., 2014; Soppina & Verhey, 2014). Phenotypically, RB2546 animals are hypersensitive to the acetylcholinesterase inhibitor aldicarb. Aldicarb has historically been used in *C. elegans* to assay both presynaptic and postsynaptic cholinergic signaling (Oh & Kim, 2017). Reduced sensitivity to aldicarb can indicate reduced presynaptic acetylcholine release or postsynaptic acetylcholine receptor defects. Conversely, hypersensitivity to aldicarb can indicate increased presynaptic acetylcholine release, increased acetylcholine receptor activity, or reduced acetylcholinesterase levels in the synaptic cleft. Our previous analysis of *klp-4 ( ok3537 )* mutants suggested that the aldicarb hypersensitivity in these animals is a result of increased neuronal traffic and ultimately increased presynaptic acetylcholine release (Magaletta et al., 2019).

To begin our analysis, we first treated both wild-type N2 and RB2546 worms with the microtubule polymerization inhibitor colchicine only, aldicarb only, or colchicine in combination with aldicarb. Colchicine treatment alone had no effect on N2 or RB2546 animals (Panel A, orange lines). However, colchicine and aldicarb in combination made N2 worms significantly more resistant to the effects of aldicarb alone (Panel A, solid blue and green lines, p<0.001, N2 +A+C *n* =135, N2 +A *n* =141). Colchicine treatment alone or colchicine with aldicarb had no effect on RB2546 worms (Panel A, dashed blue and green lines, log rank test *ns,* RB2546 +A+C *n* =167, RB2546 +A *n* =138). Since colchicine desensitizes N2 animals to aldicarb, our results suggest that presynaptic cholinergic signaling is indeed a microtubule dependent process.

Since inhibiting microtubule polymerization affected aldicarb sensitivity, we wanted to know if artificially stabilizing microtubules would have the opposite effect. Therefore, we performed similar experiments using the microtubule stabilizer taxol in conjunction with aldicarb. Taxol treatment alone or taxol and aldicarb in combination had no effect on the aldicarb sensitivity of N2 worms (Panel B, solid blue and green lines, log rank test *ns,* N2 +T *n* =196, N2 +A+T *n* =224). As hypothesized, treatment of RB2546 worms with taxol and aldicarb further exacerbated aldicarb sensitivity as compared to aldicarb treatment alone (Panel B, dashed blue and green lines, p<0.001, RB2546 +A+T *n* =135, RB2546 +A *n* =136). Thus, hyperstabilized microtubules result in increased KLP-4 mediated acetylcholine presynaptic load. Furthermore, we did not observe an effect of taxol and aldicarb in N2 animals (Panel B, solid green and blue lines, log rank test *ns* , ( N2 +A *n* =205, N2 +A+T *n=* 224)). This data suggests there exists a dynamic feedback mechanism carefully balancing microtubule stability and KLP-4 mediated cellular activities.

Microtubules are endogenously stabilized by post-translational modifications (PTMs), the most well understood of which is α-tubulin K40 acetylation (Eshun-Wilson et al., 2019). *C. elegans* contain two α-tubulin acetyltransferases; *mec-17* and *atat-2* (Cueva et al., 2012; Teoh et al., 2022) *.* To understand the role of acetylation-based microtubule stabilization on KLP-4 mediated cholinergic signaling, we obtained acetyltransferase-null mutants of both *mec-17 ( mec-17 ( ok2109 ))* and *atat-2 ( atat-2 ( ok2415 ))* . We the crossed these acetyltransferase null mutants individually with *klp-4 ( ok3537 )* mutants and assessed the aldicarb sensitivity of these new strains. *mec-17* and *atat-2* single mutants have similar aldicarb sensitivities to N2 worms (Panels C and D, solid orange and green lines, log rank test *ns,* N2 +A *n* =190, RB1696 +A *n* =161, RB1869 +A *n* =155). However, only the *atat-2* null mutation was able to rescue the aldicarb hypersensitivity phenotype seen in *klp-4* mutants (Panels C and D dashed purple and blue lines, p<0.001 for Panel D, RB2546 *n=* 162 *,* PVX69 *n* =167). Therefore, there is a specific genetic interaction between *klp-4* and *atat-2 ,* but not between *klp-4* and *mec-17* . Given that the *atat-2 ( ok2415 )* allele used here contains a significant deletion in its acetyltransferase domain we conclude that this genetic interaction represents at least part of the mechanism balancing microtubule modification and potentially stabilization and motor behavior.

If *atat-2* is indeed required for the microtubule stabilization that helps regulate KLP-4 mediated cholinergic signal in *C. elegans* , then artificially stabilizing microtubules with taxol should revert *klp-4 , atat-2* double mutants back to the *klp-4* single mutant aldicarb hypersensitivity phenotype. When we performed these experiments, we indeed observed this phenomenon; taxol treatment significantly resensitized *klp-4 ; atat-2* double mutants to aldicarb treatment similar to the *klp-4 ( ok3537 )* mutant phenotype. (Panel E dashed purple vs solid green lines, p<0.005, PVX69 +A *n=* 219, PVX69 +A+T *n* =224) *.* This data further suggests a specific genetic interaction between *klp-4* and *atat-2 in vivo* .

Our data highlight an interesting and underexplored relationship between Kinesin-3 motor function and microtubule acetylation *in vivo* . Constitutively active kinesin motors provide useful tools for uncoupling the mechanisms of kinesin regulation via autoinhibition and/or cargo binding from other kinesin-dependent cellular processes. Although we cannot definitively conclude that the *klp-4 ( ok3537 )* allele utilized in this study is a constitutively active motor both our previous studies and data contained here support this hypothesis. We previously demonstrated that *klp-4 ( ok3537 )* mutants display and increased number and a disorganization of RAB-3 positive synapses consistent with phenotypes of other Kinesin-3 gain of function alleles (Cong et al., 2021; Magaletta et al., 2019). In this study, treatment of *klp-4 ( ok3537 )* animals with aldicarb and colchicine failed to rescue the hypersensitivity phenotype of these animals. When taken together, these data further support that the *klp-4 ( ok3537 )* allele likely codes for a constitutively active motor and that this motor can overcome the effects of reduced microtubule polymerization (Panel A). Furthermore, our complementary experiments using the microtubule stabilizing drug taxol demonstrate the necessity to balance motor traffic to maintain homeostatic levels of cholinergic signaling (Panel B). Therefore, we hypothesize that there exist compensatory cellular mechanisms carefully balancing both motor activity and microtubule stability. Since microtubule acetylation is strongly implicated in the stabilization of polymerized microtubules, we used this likely overactive version of KLP-4 to isolate the role of tubulin acetyltransferase activity in this potential mechanism. We found that *atat-2* null *,* but not *mec-17* null alleles were able to rescue aldicarb hypersensitivity in the *klp-4 ( ok3537 )* background, and that taxol can eliminate the effect of *atat-2* null mutation. (Panels C, D, E). Our data points to microtubule acetylation having a key role in regulation of kinesin mediated signaling processes.

Previous studies in *C. elegans* also specifically implicate ATAT-2 tubulin acetyltransferase activity as part of the synaptic maintenance program, producing phenotypes similar to those seen in constitutively active/gain of function Kinesin-3 mutants (Borgen et al., 2019). In addition, others have also identified non-enzymatic roles of both worm tubulin acetyltransferases in synaptic branching (Teoh et al., 2022). Together, these data begin to uncover potential relationships between kinesin motors, microtubule acetylation, and biochemical interactions in both neuronal development and signal maintenance.

Although our data clearly suggests a genetic interaction between *klp-4* and *atat-2 ,* yet there remain questions on the dynamics of this relationship such as, how does microtubule acetylation affect kinesin landing and processivity, especially *in vivo* ? *In vitro* studies have demonstrated increased Kinesin-1 engagement with acetylated microtubules, suggesting acetylation levels might enhance kinesin-mediated traffic *in vivo* as well. (Reed et al., 2006). Whether this acetylation-kinesin engagement paradigm exists specifically for Kinesin-3 motors, like KLP-4 , remain less clear. Additionally, kinesin-microtubule interactions might be PTM, motor superfamily, or even cell type specific *.* For example, in *C. elegans* polyglutamylation, not acetylation, increases localization of the related Kinesin-3, KLP-6 , to ciliary microtubules (O'Hagan et al., 2011). This disparity leads to the intriguing possibility combinatorial system where different, but related motors interact with the dynamic PTMs of microtubules dependent upon the subcellular needs of the cell in a temporal manner.

## Methods

*C. elegans strains and husbandry.* All strains were maintained using standard protocols as outlined by Brenner (Brenner, 1974). All strains were originally obtained from the *Caenorhabditis* Genetics Center (CGC, University of Minnesota-Twin Cities, St. Paul MN), funded by the NIH Office of Research Infrastructure Programs (P40 OD010440) and deposited in the CGC via (Barstead et al., 2012). Genetic data on all strains were cross referenced via Wormbase (Sternberg et al., 2024). A list of strains utilized in this study can be found in Table 1. All strains obtained or constructed via crosses were verified via PCR and sequencing (Eurofins Americas, Louisville, KY).

*Aldicarb behavior assays.* Aldicarb (Millipore-Sigma, 0.5 mM final concentration), colchicine (Millipore-Sigma, 1.0 mM final concentration), and/or taxol (Millipore-Sigma, 5.0 mM final concentration) were added to normal growth media (NGM) agar plates during plate pouring (Chalfie & Thomson, 1982; Zubovych et al., 2006). All NGM plates containing drugs were used for assays within 48 hours of plate preparation. At least fifteen animals were placed on plates contain drug or drugs and a zero-time point was taken immediately. Worm paralysis was then monitored every 30-minutes for 180 minutes. Paralysis was determined by animal reaction when probed with a worm pick. Each assay was performed in triplicate, on three independent days. Data obtained from all aldicarb assays was compiled and used to construct Kaplan-Meier curves using Microsoft Excel. Log-rank analysis was performed on aggregate data from triplicate-triplicate samples (9 assays in total) to test for significance using Excel. When appropriate, p-values and final *n* values are indicated in the text.

## Reagents

**Table 1. *C. elegans* strains utilized in this study**

**Table:** 

**Strain Designation**	**Genotype**	**Source**
N2	Wild type	CGC
RB2546	*klp-4 ( ok3537 ) *	CGC
RB1696	*mec-17 ( ok2109 ) *	CGC
RB1869	*atat-2 ( ok2415 ) *	CGC
PVX64	*klp-4 ( ok3517 ), mec-17 ( ok2109 ) *	Via cross of RB2546 and RB1696
PVX69	*klp-4 ( ok3537 ), atat-2 ( ok2415 ) *	Via cross of RB2546 and RB1869
